# Using Google Trends to assess the impact of Global Public Health Days on online health information-seeking behaviour in Arabian Peninsula

**DOI:** 10.1186/s42506-020-00063-w

**Published:** 2021-02-17

**Authors:** Aymane Ajbar, Thomas A. Shepherd, Michelle Robinson, Christian D. Mallen, James A. Prior

**Affiliations:** 1 Royal College of Surgeons in Ireland, Medical University of Bahrain, Busaiteen, Bahrain; 2 School of Primary, Community and Social Care, Keele University, Keele, ST5 5BG UK; 3 Midlands Partnership NHS Foundation Trust, Stafford, UK

**Keywords:** Public Health Campaigns, Google Trends, Diabetes mellitus, Mental health, Heart disease, Hypertension, Arabian Peninsula, Global Health, Infodemiology

## Abstract

**Background:**

Global Public Health Days (GPHD) are public health interventions which serve to improve public awareness of specific health conditions. Google Trends is a publicly available tool that allows the user to view the popularity of a searched keyword during a specified time period and across a predetermined region. Our objective was to use Google Trends to assess the impact of four GPHD (World Heart Day, World Mental Health Day, World Diabetes Day and World Hypertension Day) on online health information-seeking behaviour (OHISB), 4 weeks before and a week after the GPHD, across six countries of the Arabian Peninsula (Bahrain, Kuwait, Oman, Qatar, Saudi Arabia and United Arab Emirates).

**Methods:**

Relative Search Volume (RSV) was extracted for the aforementioned countries from 28 days before the GPHD and 7 days afterwards. Statistical analysis, undertaken using joinpoint regression software, showed that GPHD have significant changes for Saudi Arabia (Diabetes, Mental Health and Heart day) and UAE (Mental Health day) but were short-lived with a fall in RSV of up to 80% after peak interest.

**Conclusion:**

GPHD appears to be effective in some countries while further research is needed to investigate the reason of its limitations.

## Introduction

Global Public Health Days (GPHD) are awareness events for a wide range of health conditions. GPHD have been defined as “brief exposure, high visibility programmes designed to stimulate thinking and discussion of certain health risks and issues by large numbers of people” [1, 2]. They serve the purpose of raising awareness of health conditions among the general public, aiming to empower them with knowledge that may be beneficial for the management and prevention of these conditions. Such campaigns are undertaken in the Arabian Peninsula (consisting of Bahrain, Kuwait, Oman, Qatar, Saudi Arabia, United Arab Emirates (UAE) and Yemen). However, the effectiveness of public health initiatives in such countries can be difficult to quantify without bespoke, large scale data collection, which is costly, time consuming and often limited in terms of geographical scope [1, 3].

Non-communicable diseases (NCD) have increased in the Arab world in the recent decades [4]. However, most Arab governments have not placed sufficient public health strategies to tackle such conditions and in general have a weak implementation of such strategies [4]. The countries of the Arabian Peninsula (with the exception of Yemen) have a high GDP per capita but are considered as developing countries and still face a number of health burdens [5]. For example, in high-income countries such as Saudi Arabia, the mean rank of deaths attributable to ischemic heart disease has increased by 7.5 from 1990 to 2010 [4]. Diabetes is also a serious chronic disease with increasing health burden in this region. For example, in Bahrain, the prevalence of diabetes mellitus rose from 17.3% in 2013 to 21.8% in 2014 [6]. Mental illness is another significant health problem, particularly among students in the region. It was shown that the rate of mental illness in Saudi high school students is around 48% [7]. Ischemic heart disease is the most common cause of death in certain regions in the Arabian Peninsula and hypertension is a major risk factor for developing cardiovascular events, kidney disease and blindness. The age-standardised rate for hypertension in a sample from the United Arab Emirates was reported to be 33% [8].

Countries of the Arabian Peninsula have experienced a dramatic increase in internet usage over the past decade, where the increasing trend is higher than the rest of the world [9] and globally the internet service growth rate was 10 times higher in Saudi Arabia [10]. Bahkali et al. [11] found that there is a high interest among the Arab world in seeking online health information. GPHD have the potential to increase online health information-seeking behaviour (OHISB) among the Arab population and may be used as a powerful long-term strategy for promoting public health. However, despite the relatively high costs and frequency of their implementation, there is little evidence in the literature examining the effectiveness of GPHD [1]. Google Trends is used in many areas in health research [12]. For example, Schootman et al. [13] used Google Trends to assess interest in cancer screening in the USA. The use of Google Trends allows for a cheaper, faster alternative to large data collection for testing the effectiveness of public health campaigns [3] and has been previously used to assess the impact of GPHD on OHISB [14]. The aim of this study is to assess the effect of Global Public Health Days (World Heart Day, World Mental Health Day, World Diabetes Day and World Hypertension Day) on online health information-seeking behaviour in countries of Arabian Peninsula (Bahrain, Kuwait, Oman, Qatar, Saudi Arabia, United Arab Emirates) [15].

## Methods

### Google Trends

Google Trends (GT; Alphabet Inc., Mountain View, CA, USA) is a free to use, publicly available online resource provided by Google [16]. GT shows the popularity of keywords used over a given time period and within a defined locality. It collects search data which is representative of all Google searches, with popularity of a keyword determined by the Relative Search Volume (RSV). The RSV is calculated by dividing the sampled search data points by the total number of searches done in the specific region and time, after which the resulting value is scaled from 0 to 100 [17]. GT only shows the searches performed by users, not their activity afterwards, and excludes searches from the same individual over a short period of time. The data collected by GT is anonymous and cannot be linked to a single individual.

### Search term input

In order to ensure the quality and reproducibility of the GT data, we used the checklist provided by Nuti et al. [12]. A component of the checklist is the rationale for search input and settings. The chosen GPHD were World Diabetes Day, World Mental Health Day, World Heart Day and World Hypertension Day. These GPHD were chosen based on local recognition (the presence of GPHD in the government sites and newspapers), prevalence, and importance of the disease burden they represent in the region, in terms of both morbidity and mortality. Search terms were selected to reflect the disease names campaigned by the GPHD. Therefore, the search terms “Mental health”, “Heart disease”, “Diabetes” and “Hypertension” were used for the corresponding GPHD. When a search term is entered in GT, a “search category” is chosen. A search category includes all search term variations and incorporates different languages. This is particularly important as Arabic is the predominant language used in the Arabian Peninsula. It also allows the inclusion of languages spoken by expatriate populations, such as Tagalog. Search categories also allow for related search terms to be included in the data. For example, searching for “Heart Disease” (topic) allows for both the Arabic translation and other related search terms such as “coronary artery disease” to be included, as well as other synonyms. Table 1 shows the search input and the corresponding Arabic terms included. Query categories have been left as “All categories” as the categories “Health” and “Science” were separated. This is important because an individual may search for both clinical and scientific facts on the health burden which may be filtered out if one category is chosen over the other.

**Table 1:** Global Public Health Days search category and corresponding Arabic translations

Search Category (English)	Included term (Arabic)
Mental Health (Topic)	النفسية الصحة
Heart Disease (Topic)	القلب امراض
Diabetes (Disorder)	السكري مرض
Hypertension (Medical Condition)	الدم ضغط ارتفاع

The Arabian Peninsula consists of Bahrain, Kuwait, Oman, Qatar, Saudi Arabia, United Arab Emirates and Yemen. All these countries are similar in terms of local cultures. However, Yemen has a much smaller GDP per capita than the other countries, lower internet penetrance, and it is in a civil war [18, 19]. Therefore, it was excluded from our analysis. The internet penetration for the remaining chosen countries was as follows: Bahrain (98.6%), Kuwait (99.6%), Oman (80.2%), Qatar (99.7%), Saudi Arabia (93.3%) and United Arab Emirates (98.5%) [20].

### Search dates

**Fig. 1:**
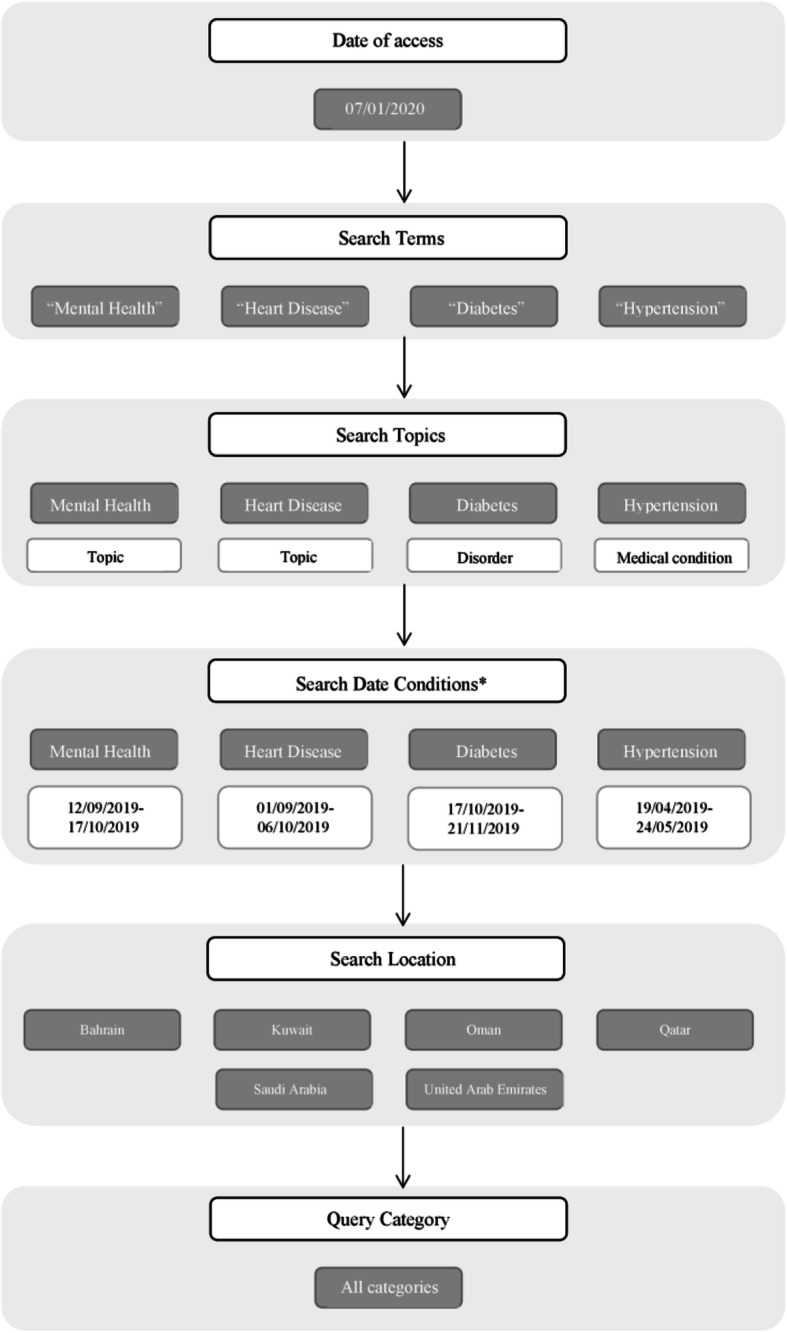
Flowchart depicting the search methodology. *Search Dates include a total of 36 days, 28 days before global public health day and 7 days after

Another component of the checklist is the time period which was searched. Search conditions included data from 28 days before the GPHD and up to 7 days after; a total of 36 days. This is to ensure that data is captured relating to increased OHISB prior to the GPHD and to observe any short-term changes to the trend afterwards that can be attributed to the GPHD. The dates for GPHD were based on local dates obtained from the Ministries of Health of the respective countries. If this data was not available, other sources such as newspapers and regional university websites were checked. If no dates, or conflicting dates were found, the date was assumed to be the international recognized date for that condition. A flowchart depicting the process used in our study along with the search inputs used is presented in Fig. 1.

### Data analysis

Another component of the checklist is the access date. GT was accessed and RSV data downloaded as a comma-separated values (.csv) file on January 7th 2020. The data was analysed statistically using the Joinpoint Regression Program, which was developed by the National Cancer Institute [21]. This software analyses trends by regression modelling while searching for joinpoints. The analysis of regression lines aims to discover when a trend changes (joinpoints) and estimates the regression function from previous joinpoints [22]. The .csv file was downloaded and appropriate parameters were set within the software. The independent variable was the Day ID, where the 29th of the 36 days represented the GPHD and the dependent variable was set to be the RSV value. The model assumed that the errors have a constant variance, and the number of joinpoints was set for a minimum of zero and a maximum of three [15]. The minimum number of observations from a joinpoint to either end of data was set to two and the minimum number of observations between joinpoints was also set to two. No logarithmic transformation was applied to the data. For countries without trends, an average RSV value was calculated across the time period of 36 days [15]. One-way analysis of variance (ANOVA) was used to assess any significant differences in the mean of RSV values between the selected countries for each GPHD.

## Results

**Fig. 2:**
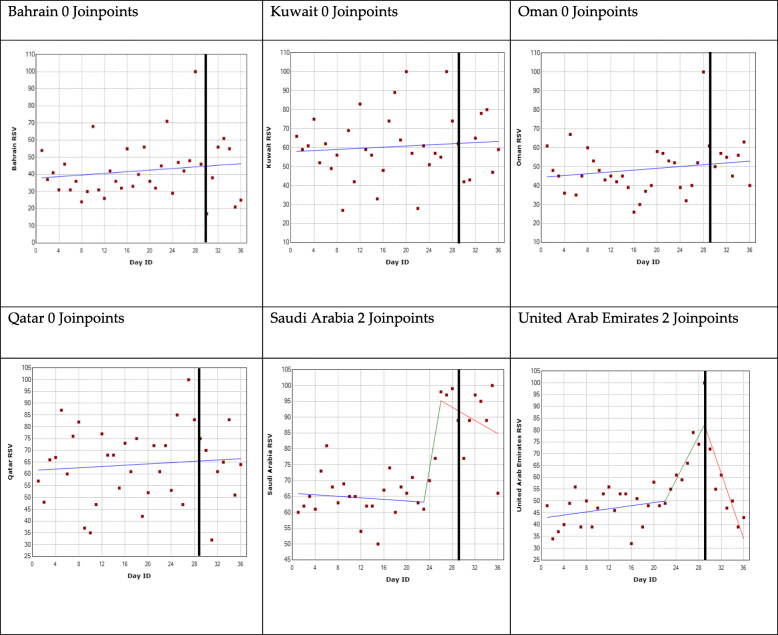
World Diabetes Day joinpoint regression analysis. *The black vertical line represents the global public health day

**Fig. 3:**
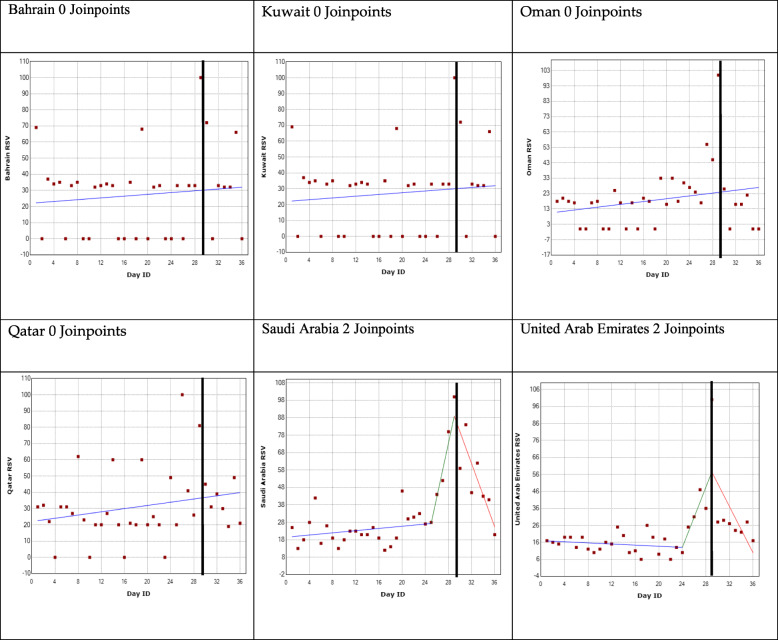
World Mental Health Day joinpoint regression analysis. *The black vertical line represents the global public health day

**Fig. 4:**
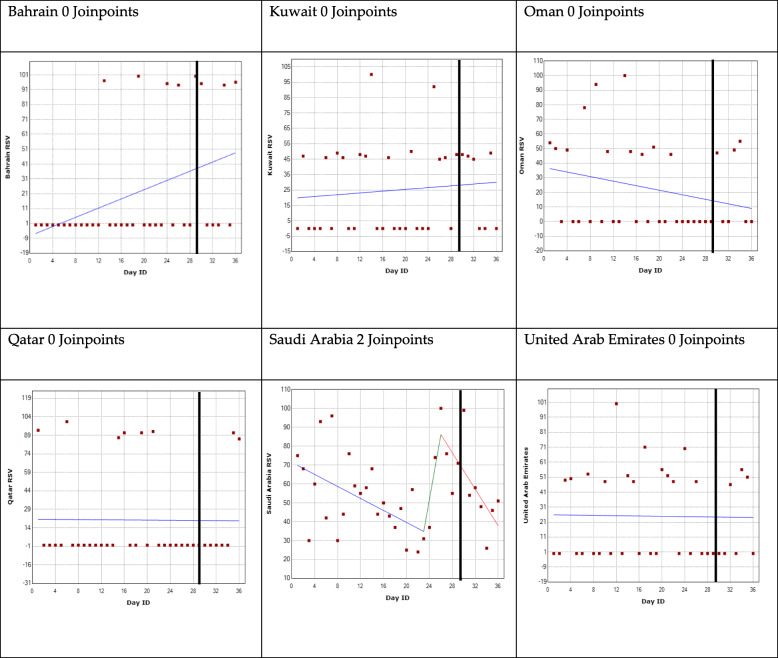
World Heart Day joinpoint regression analysis. *The black vertical line represents the global public health day

**Fig. 5:**
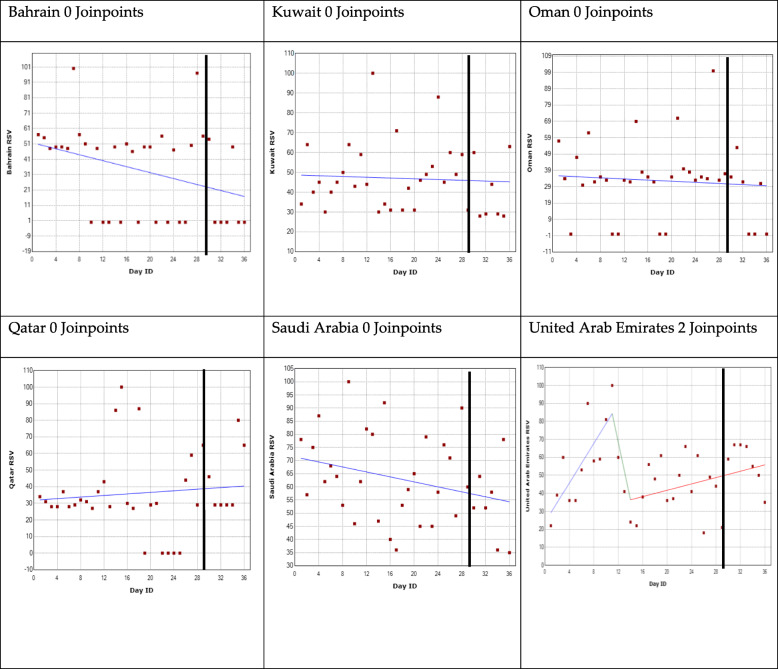
World Hypertension Day joinpoint regression analysis. *The black vertical line represents the global public health day

The results are represented for each country and for each GPHD using a regression model shown in Figs. 2, 3, 4 and 5. The GPHD is represented in the graph by the black vertical line. The average RSV values for each country across the 36-day period is provided in Table 2. One-way ANOVA revealed that there is a statistically significant difference (*p* < 0.05) among the RSV means for each GPHD.

**Table 2:** Average relative search volume values for the selected countries of Arabian Peninsula for each Global Public Health Day

	Bahrain	Kuwait	Oman	Qatar	Saudi Arabia	United Arab Emirates	*P* value
Diabetes	42	61	49	64	73	52	< 0.001
Mental health	27	27	20	31	34	21	0.02
Heart	21	25	23	20	56	25	< 0.001
Hypertension	34	47	33	36	63	50	< 0.001

### World Diabetes Day

The analysis for Bahrain, Kuwait, Oman and Qatar identified no joinpoints, indicating that there was no change in OHISB either pre or post the GHPD. The average RSV value from 28 days before the GPHD and up to 7 days afterwards was 42, 61, 49 and 64 for Bahrain, Kuwait, Oman and Qatar respectively.

The analysis for Saudi Arabia and United Arab Emirates revealed two joinpoints. Saudi Arabia showed the first joinpoint a week before the GPHD, after which there was a sharp increase in RSV. The second joinpoint was three days prior to the GPHD after which there was a decrease in RSV. Following the 7 days leading up to the GPHD, there was a 41% increase in RSV, peaking at 99 a day before the GPHD. It then declined to 66 a week afterwards, but this remained higher, on average, than pre-GPHD. For UAE, the first joinpoint also occurred a week before the GPHD, but the second joinpoint occurred on the day of the GPHD, after which there was a decreasing trend. Following the 7 days leading up to the GPHD, there was a 104% increase in RSV peaking at 100 on the day of the GPHD. It then declined to 43 a week afterwards (Fig. 2).

### World Mental Health Day

The analysis for Bahrain, Kuwait, Oman and Qatar identified no joinpoints. The average RSV value from 28 days before the GPHD, up to 7 days afterwards, was 27, 27, 20 and 31 for Bahrain, Kuwait, Oman and Qatar, respectively.

The analysis for Saudi Arabia and United Arab Emirates revealed two joinpoints. Both showed the first joinpoint 4 days before the GPHD with a rise in trend, while the second joinpoint occurred on the day of the GPHD, after which, there was a declining trend. For Saudi Arabia, following the 7 days leading up to GPHD, there was a 223% increase in RSV peaking at 100 on the day of the GPHD. It declined to 21 a week afterwards. For UAE, following the 7 days leading up to GPHD, there was a 1567% increase in RSV peaking at 100 at the day of the GPHD. It declined to 17 a week after (Fig. 3).

### World Heart Day

The analysis for Bahrain, Kuwait, Oman, Qatar and UAE identified no joinpoints. The average RSV value from 28 days before the GPHD up to 7 days afterwards was 21, 25, 23, 20 and 25 for Bahrain, Kuwait, Oman, Qatar and UAE, respectively.

The analysis for Saudi Arabia revealed two joinpoints. The first joinpoint was a week before the GPHD with a rise in trend until 3 days prior to the GPHD, where the second joinpoint occurred after which there was a decreasing trend. Following the 7 days leading up to GPHD, there was a 317% increase in RSV peaking at 100, 3 days before the GPHD. It declined to 80 a week afterwards. The joinpoint regression graphs are shown in Fig. 4.

### World Hypertension Day

The analysis for Bahrain, Kuwait, Oman, Qatar and Saudi Arabia identified no joinpoints. The average RSV value from 28 days before the GPHD up to 7 days afterwards was 34, 47, 33, 36 and 63 for Bahrain, Kuwait, Oman, Qatar and Saudi Arabia, respectively.

The analysis for United Arab Emirates revealed two joinpoints. The first joinpoint occurred 18 days before the GPHD, after which, there was a falling trend. The second joinpoint occurred 3 days after the first joinpoint, after which, there was a rising trend. The first joinpoint occurred when RSV was 100. After the first joinpoint, RSV fell by 76% until the second joinpoint reaching a value of 24. After the second joinpoint, RSV increased to 35 a week after the GPHD (Fig. 5).

## Discussion

We examined the role of several GPHD on the OHISB in countries across the Arabian Peninsula. We found that only searches performed in Saudi Arabia and UAE showed statistically significant changes before and after the public health day and that these trends varied by condition and country.

The UAE and Saudi Arabia showed a significant change in trend towards Mental Health day. This aligns with the latest burden data which shows both of these countries having a relatively high DALY for mental illness [23]. Furthermore, both UAE and Saudi Arabia also showed trends in diabetes day, and in Saudi Arabia, this trend remained higher than pre-GPHD levels, suggesting that of all the GPHD, this one may be resonating with this population. Despite having the lowest DALY, Saudi Arabia was among the top 10 countries with the highest prevalence of diabetes as of 2013 [24]. Furthermore, GT data from Saudi Arabia also showed a change in trend for heart day. This aligns with burden data showing that Saudi Arabia has the 3rd highest DALY among the countries in our study for cardiovascular disease. Only UAE showed a trend with hypertension day, even though hypertension is a major risk factor for disease in most of our studied countries [23]. This may be due to a lack of awareness of the health day in the other countries. Both Saudi Arabia and UAE have shown increasing OHISB in the build up towards their respective health diabetes day. The change in trend was significant which implies that diabetes day has an impact on online health information-seeking behaviour in these countries. Despite diabetes being a very significant health burden in most Arabian Peninsula, especially in Bahrain, there was no significant trend found in most of these countries. This is also true for other health burdens chosen in this study. It should be noted that the reasons GPHD are more effective in Saudi Arabia and UAE may be due to (1) a higher DALY, (2) a higher population and (3) a higher awareness of the health day itself.

Wherever a significant rise in trend was found, there was a fall shortly afterwards which indicates that the effect of GPHD was short lived (though to a lesser degree for diabetes day in Saudi Arabia). This has important implications as to the efficacy of GPHD in the region, and there may be multiple reasons for the decline in internet activity. These could be positive, with the potential that the health day may have prompted people to search support from their healthcare provider, as opposed to searching in Google. Another reason could be that the awareness was more widespread in social media sites after the health day, thereby users could have sought online health information from the use of social media sites, such as Twitter. It was shown that social media has a significant influence on health-seeking behavior in Saudi Arabia [25] and that women in Saudi Arabia were more interested in discussing gynaecological conditions on Twitter [26]. This may suggest a preference of use of social media over search engine sites, thereby giving a lower observed trend. However, it is likely that the GPHD is in fact short-lived and users lose interest in the health problem after the health day.

Reasons for the lack of trends in some of the studied countries could be due to poorer data collection from Arabian countries with smaller populations such as Bahrain [27, 28]. In fact, no trend has been seen with Bahrain even though it has a higher health-care GDP spending than any of the other countries (excluding Saudi Arabia) [29]. In addition, the GPHDs studied may not be “popular” in this region, although all health days included were present as official events in UAE, Bahrain and Saudi Arabia’s government website. The governments could have played a role in promoting the health day, thereby explaining the trend seen in Saudi Arabia and UAE. Also, GT may not reflect the disease burden of a country. Alicino et al. [30] showed that the relation between GT and countries affected by the Ebola outbreak was weak to moderate.

GPHD were shown to raise public awareness of disease and can raise awareness on symptoms, such as “Act FAST” campaign for Stroke symptoms [31]. However, though GPHD have been shown to induce behaviour change [1], campaigns promoting health have yielded only short-term improvements in health behaviour, as reflected in this study, demonstrating that behaviours are difficult to sustain after a campaign ends [3]. It was shown previously that public health campaigns can result in improved clinical outcomes (reduction in mortality from cardiac arrests and reduction in patient delays for strokes) [32, 33]. Therefore, public interventions such as GPHD can play a key role in preventing disease and improving clinical outcomes among the general population. The type of campaign promoted by GPHD can influence its success. Snyder et al. [34] showed that campaigns which encourage new behaviour have a greater effect than cessation campaigns. GPHD success is multifactorial and its outcome on an individual is influenced by many determinants, including physical, social and economic environment [35]. Addressing social pressure is a key element for success of a campaign [36]. Zolezzi et al. [37] showed that there is large diversity in stigmatizing beliefs in the Arab world towards mental illness, with Qatari population showing more of these beliefs than non-Qatari Arabs. Longevity of a campaign can also affect its success. A public health campaign should be planned to last as long as it takes to achieve an outcome [38]. GPHD are aimed at the general population. However, a key element for success of a health campaign is to define a target population [36]. GPHD should be promoted in media used by the target audience. For example, ischemic heart disease may be more prevalent among the elderly in the population of the countries we have examined; therefore, GPHD should be conveyed to them through routes that can reach them, such as public awareness campaigns as opposed to internet-based media. Addressing socioeconomic disparities in Arab countries can influence the effectiveness of public health campaigns. Donnelly et al. [39], for instance, showed that low socioeconomic status among Middle Eastern women impedes their participation in breast cancer screening.

### Strengths and limitations

The search engine market share in Saudi Arabia as of 2018-2019 was 97.38% while that for other countries was no less than 95% indicating that Google is used primary as a search engine in the Arabian population making our results more generalizable. Furthermore, the use of search categories allows us to include searches performed in any language. Also, the choice of “All categories” would include any search regardless of its category. Finally, the use of the checklist produced by Nuti et al. [12] improved the quality and reproducibility.

The use of GT for health research is still in its infancy and care should be taken to reduce its limitations [40]. One of which is that GT methodology is publicly unknown. The extracted data showed a high variability in RSV with some countries showing alternating zero values (suggesting no searches undertaken on that day). There could be that a significant proportion of users were searching for the medical condition during those dates, however, GT labelled low volume data relative to the peak as zero [17]. Another limitation for using GT is that data is only collected from those people with internet access and those who use Google. Therefore, it would not be reflected in our study if the GPHD raised awareness among low-income groups or novice computer users. Finally, the wide variability in the data may have resulted in the statistical software incorrectly identifying joinpoints.

## Conclusions

Our study highlighted the limited effects of GPHD on OHISB in six countries of the Arabian Peninsula. This raises the question of the effectiveness of such intervention in the region. These results could imply that the effect of public health interventions in general is poor in the region. It is also imperative for local researchers to further explore the reasons for the low or non-existent response of the population to GPHD that aimed at improving awareness on significant health burdens in the region. Although GT can be a powerful tool for public health surveillance, documentation of its methods and assumptions should be released to allow for transparency and reproducibility.

## Data Availability

The datasets used and/or analysed during the current study are available from the corresponding author on reasonable request.

## Abbreviations

GPHD: Global Public Health Days
OHISB: On online health information-seeking behaviour
RSV: Relative Search Volume
GT: Google Trends
UAE: United Arab Emirates
